# Association Between Post-Operative Infection and Blood Transfusion in Cardiac Surgery

**DOI:** 10.7759/cureus.8985

**Published:** 2020-07-03

**Authors:** Abdulkarim W Abukhodair, Mohammed S Alqarni, Ziad M Bukhari, Ammar Qadi, Hani N Mufti, Jose Andres Fernandez, Sean R Bennett

**Affiliations:** 1 Medicine, King Saud Bin Abdulaziz University for Health Sciences, Jeddah, SAU; 2 Cardiac Surgery, King Faisal Cardiac Center, King Abdullah Medical City, Jeddah, SAU; 3 Medicine, King Abdullah International Medical Research Center, Jeddah, SAU; 4 Anesthesiology, King Faisal Cardiac Center, King Abdullah Medical City, Jeddah, SAU

**Keywords:** blood transfusion, cardiac surgery, infection, jeddah

## Abstract

Background

Blood transfusion is a commonly used therapy in cardiac surgery, whether it is given during the surgery or in the intensive care unit. It is important to evaluate the risks and benefits of exposure to blood transfusion. The use of blood transfusions can influence patient outcome. Previous studies have implicated blood transfusion as a causative factor in post-operative infection.

Objectives

We aim to determine the effect of blood transfusion on post-operative infection in cardiac surgery patients at the King Faisal Cardiac Center, Jeddah, Saudia Arabia, from January 2017 to January 2019.

Methods

The regular six-week follow-up of cardiac surgery patients allowed us to maintain a six-week infection span. The main variables included patient characteristics, operative characteristics, pre-operative hemoglobin, six-week infection, blood transfusion, and clinical outcomes. A logistic regression model was developed to identify patient and procedure variables that were associated with blood transfusion and infection. The baseline variables were entered into the model. Variables with p-value less than 0.05 were considered significant.

Results

The incidence of transfusion out of 197 patients was 93.4% (n = 184). The occurrence of infection was 31.82% (n = 63). There was no difference in post-operative infection for patients who received blood transfusions compared with those who did not receive blood transfusions (p = 0.902). In comparing patients receiving 1-2 units of red blood cells (RBCs) (48%) and those receiving >2 units of RBCs (52%), there was no significance (p = 0.549).

Conclusions

There was no association between the incidence of infection and blood transfusion. While there are other reasons for withholding blood, it would not be recommended to do so based on the concern of infection.

## Introduction

Cardiac surgery is a very demanding intervention that is increasingly performed, and it will continue to rise due to the increasing age of the population. Surgery is used to treat many heart diseases such as coronary artery disease (CAD) and valvular disorders. CAD is a widespread and often fatal disease that is responsible for more than one-third of the deaths in individuals 35 years or older [1,2]. Both CAD and valvular disorders are commonly treated surgically which exposes patients to peri-operative complications. Numerous advancements in cardiac surgery have made it relatively safe, with a mortality and morbidity rate of 3% and 14%, respectively [3,4].

Blood transfusion is common in cardiac surgery, whether it is given during the surgery or in the intensive care unit (ICU) [5]. A study found that roughly 60% of coronary artery bypass graft surgery (CABG) patients receive a blood transfusion [6], but figures vary from 0% to 75%. Due to this variability, it is necessary to evaluate the risk and complications that a patient undergoing cardiac surgery is exposed to by receiving a blood transfusion. In an attempt to reduce such risks, several techniques have been devised to improve the storage and optimize the utilization of blood products [7]. However, an increase in infection among transfused cardiac surgery patients has been reported [8,9].

Post-operative infection in cardiac surgery patients has been linked to patient outcome [10]. Nosocomial pneumonia, surgical site infection, mediastinitis, bacteremia, and sepsis are common infectious processes that can affect outcome [11].

At King Faisal Cardiac Center, Jeddah, Saudia Arabia, the high infection and blood transfusion rates have been raising questions regarding whether or not they are associated with each other. We aim to determine the effect of blood transfusion on post-operative infection in cardiac surgery patients at King Faisal Cardiac Center from January 2016 to January 2019.

## Materials and methods

This is a cohort study using a non-probability consecutive sampling technique for selection of the study population to identify the association between blood transfusion and post-operative infection in cardiac surgery patients at King Faisal Cardiac Center. The research project was approved by King Abdullah International Medical Research Center, Jeddah, Saudi Arabia, and the Institutional Review Board.

Patients older than 18 years of age who were undergoing cardiac surgery were eligible to participate; patients currently immunosuppressed or with active systemic infections were excluded. No informed consent was needed for this study. Patients exposed to blood transfusion at any time during the ICU stay or surgery were placed in the “blood transfusion” group; patients not exposed to blood transfusion at all were placed in the “no blood transfusion” group.

Peri-operative variables and blood-component utilization forms are regularly collected along with data from the Best Care system. The regular six-week follow-up of cardiac surgery patients allowed us to maintain the six-week infection span. Using the Best Care system, data were collected at the hospital by the authors and co-authors. The main variables for the data collection sheet included patient characteristics, operative characteristics, laboratory results, infection six months post-surgery, blood transfusion, and clinical outcomes. Data will be disposed of after one year of publishing.

Post-operative mortality was defined as an in-hospital death. Infection consisted of pneumonia, sepsis, Clostridium difficile colitis, mediastinitis, deep surgical site infection (DSSI) in the chest, DSSI in the leg or groin, and myocarditis, pericarditis, and endocarditis. These infections were confirmed by culture. Blood transfusion data were collected in-depth, calculating the units transfused intra-operatively and postoperatively, including fresh frozen plasma (FFP) and platelet transfusion.

Categorical variables were summarized by frequencies and percentages, and continuous variables by means and standard deviations or by medians and interquartile ranges (IQRs) if their distributions were skewed. Baseline univariate comparisons between those who did and did not receive blood transfusion were made using Wilcoxon’s rank-sum and the chi-square tests where appropriate.

Logistic regression was used to model the probability of infection and in-hospital mortality as a function of blood transfusion. A logistic regression model was developed to identify patient and procedure variables that were associated with blood transfusion and infection. The baseline variables were entered into the model. Variables with p-value less than 0.05 were considered significant. All statistical analysis and assessment of the model’s performance were conducted using the R Software, version 3.3.0 (R Foundation for Statistical Computing, Vienna, Austria).

## Results

Our sample size included 197 patients. Participants in this study were in the age range of 18 to 84 years, with a median age of 60 years (IQR = 14 years). Body mass index (BMI) ranged from 16.22 to 50.68, with a median BMI of 28.86 (IQR = 7.22). Among our population, 71.07% (n = 140) were males. Our population presented with a high prevalence of cardiac risk factors: diabetes (71.72%; n = 142), hypertension (76.14%; n = 150), dyslipidemia (52.79%; n = 104), smoking (24.37%; n = 48), heart failure (8.63%; n = 17), and COPD (2.03%; n = 4). Refer to Table 1 for more on the patients’ characteristics.

**Table 1:** Patient characteristics SD, standard deviation; BMI, body mass index; COPD, chronic obstructive pulmonary disease; IQR, interquartile range; CABG, coronary artery bypass graft surgery; OR, operating room; PRBC, packed red blood cells; FFP, fresh frozen plasma; C. Diff, Clostridium difficile; SSI, surgical site infection; UTI, urinary tract infection

Patient Characteristic	n = 198 Patients
Pre-Operative Characteristics	
Age (years)	
Mean ± SD	58 ± 12.4
Male gender (%)	71
BMI (kg/m^2^)	
Mean ± SD	28.9 ± 5.4
Smoking (%)	24.4
Hypertension (%)	76
Diabetes mellitus, (%)	72
Dyslipidemia (%)	53
COPD (%)	2
Heart failure (%)	8.6
Pre-operative hemoglobin (gm/dL)	
Mean ± SD	12.7 ± 2.1
Pre-operative creatinine (µmol/L)	
Median ± IQR	82 ± 33
Pre-operative HbA1C (%)	
Median ± IQR	6.9 ± 3.1
Intra-Operative Characteristics	
Type of surgery (%)	
CABG	63
Valve	21
CABG + valve	10
Other	6
OR time (in minutes)	
Mean ± SD	294 ± 124
Post-operative characteristics	
Post-operative hemoglobin (gm/dL)	
Mean ± SD	8.9 ± 1.3
Post-operative creatinine (µmol/L)	
Median ± IQR	90 ± 51
PRBCs transfusion (%)	91
Platelet transfusion (%)	18
FFP transfusion (%)	22
Post-operative infection (%)	31.3
Type of infection (%)	
Pneumonia	6.7
Blood	1
C. Diff	0.5
SSI (sternum)	16
SSI (leg)	5
UTI	3
Re-operation for infection (%)	4.6
Discharge Characteristics	
Discharge hemoglobin (gm/dL)	
Mean ± SD	11 ± 1.3
Length of stay (days)	
Median ± IQR	9 ± 6
In-hospital mortality (%)	4

To carefully compare and assess the association of blood transfusions with post-operative infection, we divided the patients into two groups: transfused and non-transfused; however, due to the small number of non-transfused patients, the groups were later divided into another two groups: patients receiving more than 2 RBC units and those receiving less or equal to 2 RBC units. Factors such as age, gender, BMI, and diabetes were similar in all groups (Table 2).

**Table 2:** Univariate Analysis SD, standard deviation; NS, non-significant; BMI, body mass index; COPD, chronic obstructive pulmonary disease; IQR, interquartile range; CABG, coronary artery bypass graft surgery; OR, operating room; FFP, fresh frozen plasma; C. Diff, Clostridium difficile; SSI, surgical site infection; UTI, urinary tract infection

Patient Characteristics	RBC Transfusion > 2 Units		p-Value
	No, 52.5% (n = 104)	Yes, 47.5% (n = 94)	
Pre-Operative Characteristics			
Age (years)			
Mean ± SD	57 ± 12	58 ± 11	NS
Male gender %	83	57.5	<0.001
BMI (kg/m^2^)			
Mean ± SD	28.9 ±5.2	28.8 ±5.6	NS
Smoking (%)	30	18	0.049
Hypertension (%)	76	77	NS
Diabetes mellitus (%)	76	68	NS
Dyslipidemia (%)	59	46	0.065
COPD (%)	2.9	1.1	NS
Heart failure (%)	8.7	8.5	NS
Pre-operative hemoglobin (gm/dL)			
Mean ± SD	13.4 ± 2	12 ± 2	<0.001
Pre-operative creatinine (µmol/L)			
Median ± IQR	78.5 ±27.8	84 ±38.8	0.036
Pre-Op HbA1C (%)			
Median ± IQR	7.8 ± 2.3	7.4 ± 2.1	NS
Intra-Operative Characteristics			
Type of surgery			NS
CABG	66	61	
Valve	22	19	
CABG + valve	6	15	
Other	8	18	
OR time (in minutes)			
Mean ± SD	263 ±117	326 ±124	<0.001
Post-operative Characteristics			
Post-operative hemoglobin (gm/dL)			
Mean ± SD	9.3 ± 1.2	8.5 ± 1.2	<0.001
Post-operative creatinine (µmol/L)			
Median ± IQR	84.5 ±39.5	100 ± 93	<0.001
Platelet transfusion (%)	7.7	30	
FFP transfusion (%)	9.6	35.1	<0.001
Post-operative infection (%)	30.4	34.4	NS
Type of infection (%)			NS
Pneumonia	8.8	4.3	
Blood	0	2.2	
C. Diff	1	0	
SSI (sternum)	15.7	16.1	
SSI (leg)	2.9	7.5	
UTI	2	4.3	
Re-operation for infection (%)	2	7.5	0.064
Discharge Characteristics			
Discharge hemoglobin (gm/dL)			
Mean ± SD	10.7 ±1.3	1.7 ± 1.3	NS
Length of stay (days)			
Median ± IQR	8 ± 3	11 ± 6	0.003
In-hospital mortality (%)	2	6.4	NS

The incidence of transfusion out of 197 patients was 93.4% (n = 184). This includes RBC transfusion, platelet transfusion, and FFP. As for RBC transfusion, 35 patients (17.77%) received 1 unit, 52 (26.4%) received 2 units, 35 (17.77%) received 3 units, 34 (17.26%) received from 4 to 5 units, 25 (12.69%) received from 6 to 16 units, and 16 (8.12%) did not receive any transfusion at all.

**Figure 1:**
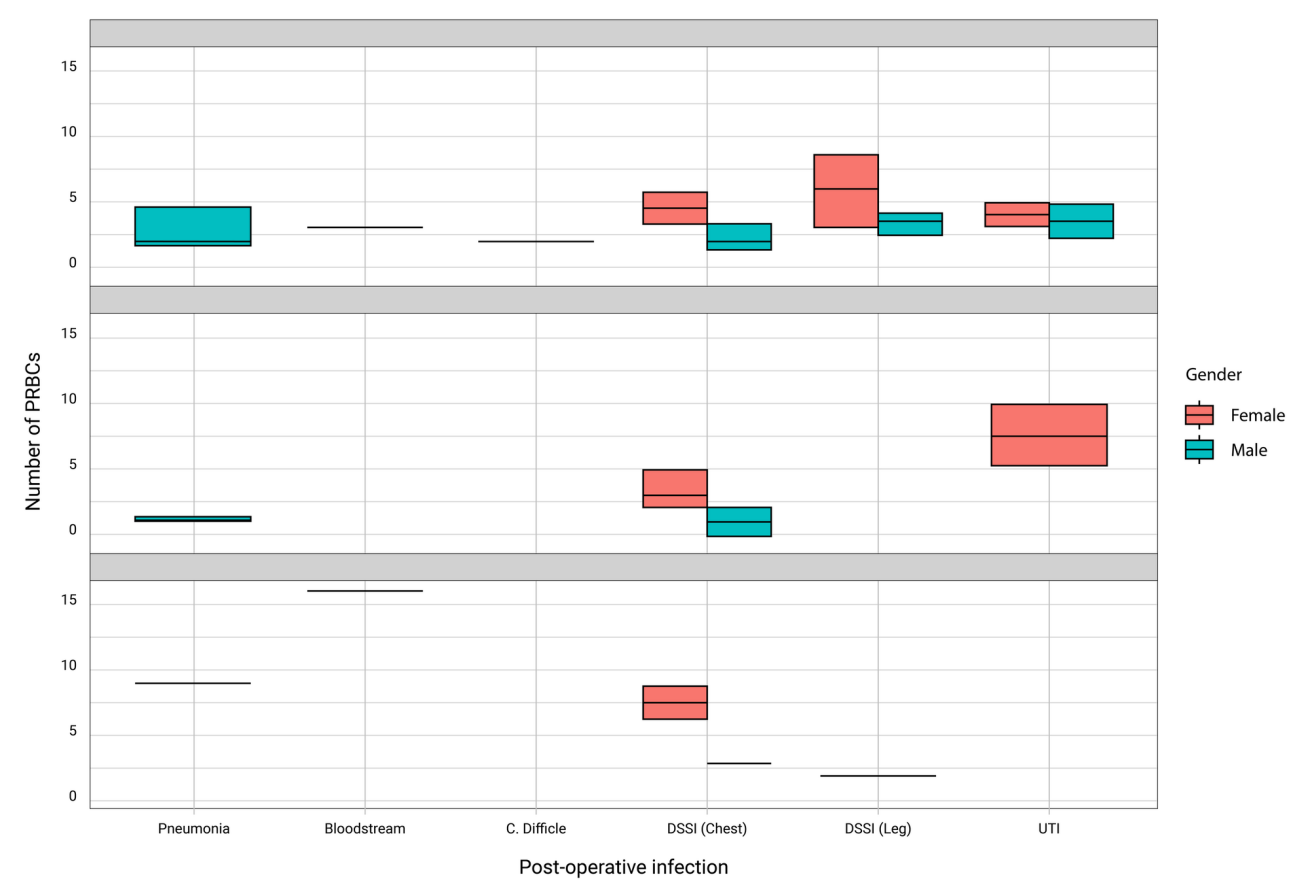
Occurrence of post-operative infection in males and females associated with the number of PRBC transfusion for different operations PRBC, packed red blood cells

**Figure 2:**
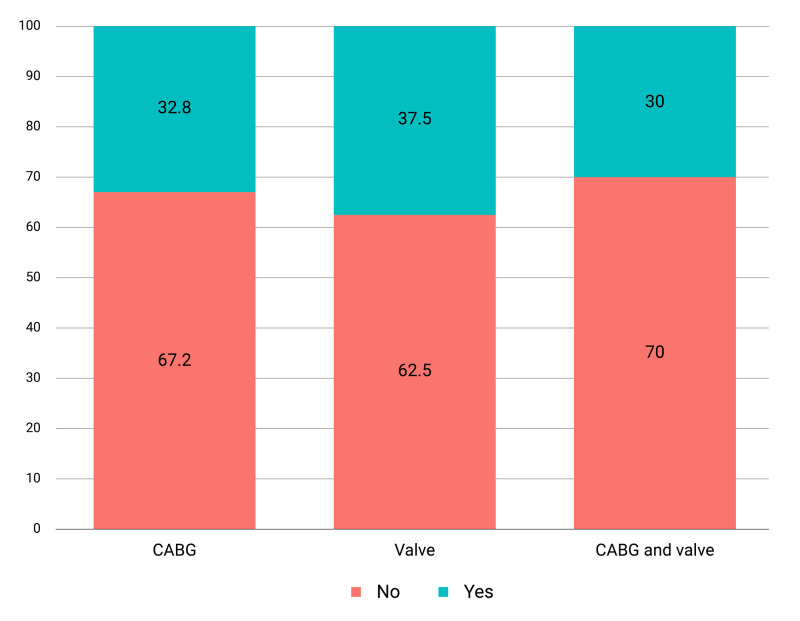
Infection by type of procedure CABG, coronary artery bypass graft surgery

The incidence of six-week postoperative infection was 31.82% (n = 63). Among those infections were pneumonia (20.63%; n = 13), bloodstream (3.17%; n = 2), C. Difficile colitis (1.59%; n = 1), DSSI (chest) (49.21%; n = 31), DSSI (leg) (15.87%; n = 10), and urinary tract infection (9.52%; n = 6) (Figure 1). The incidence of infection by type of operation is stated in Figure 2.

The incidence of transfusion was 93.3% (n = 182), and the incidence of six-month postoperative infection was 31.82% (n = 63). There was almost no difference in post-operative infection for patients who received blood transfusions as opposed to those who did not receive blood transfusions (p = 0.902). In comparing patients receiving more than 2 units of RBCs (47.5%) with patients not receiving more than 2 units of RBCs (52.5%), there was no significance (p = 0.444). There is some relationship between amount of RBCs used and wound infection after CABG, especially in women (Figure 1).

Transfused and non-transfused patients showed similar hemoglobin (Hb) values on discharge. Only pre-operative Hb was significantly different in these two groups (p < 0.001), with a lower pre-operative Hb in the transfused group.

Pre-operative, post-operative, and discharge Hb were not associated with a risk of infection. Interestingly, pre-operative heart failure and prolonged hospital stay were both significantly associated with post-operative infection (Table 3).

**Table 3:** Transfusion and Infection OR OR, odds ratio; CI, confidence interval; BMI, body mass index; DM, diabetes mellitus; HF, heart failure; HTN, hypertension; Hb, hemoglobin; PRBC, packed red blood cell

		Transfusion					Infection			
Variable		OR	CI 2.5%	CI 97.5%	p-Value		OR	CI 2.5%	CI 97.5%	p-Value
Gender, female		2.625	0.686	17.225	NS		1.431	0.746	2.718	NS
Age > 60 years		0.840	0.277	2.546	NS		1.447	0.793	2.654	NS
BMI > 30		1.195	0.396	4.019	NS		0.981	0.528	1.805	NS
Operating room time > 270 minutes		6.621	1.742	43.317	0.015		0.654	0.354	1.194	NS
Smoking, yes		2.015	0.524	13.255	NS		0.543	0.246	1.124	NS
Type II DM, yes		1.478	0.436	4.495	NS		1.983	0.981	4.249	NS
HF, yes		0.536	0.129	3.646	NS		3.369	1.229	9.730	0.019
HTN, yes		1.865	0.548	5.709	NS		1.988	0.942	4.517	NS
Dyslipidemia, yes		1.537	0.514	4.836	NS		1.294	0.708	2.381	NS
Mortality, yes		0.517	0.083	10.044	NS		0.689	0.099	3.089	NS
HbA1c > 7		2.177	0.683	8.267	NS		1.701	0.931	3.138	NS
Hospital stay > 9 days		1.709	0.536	6.492	NS		3.250	1.754	6.134	0.000
Pre-operative Hb ≤ 10		0.952	0.000	Inf	NS		1.621	0.660	3.864	NS
Post-operative Hb > 10		0.230	0.070	0.816	0.016		0.865	0.339	2.038	NS
Pre-operative creatinine >82		3.444	1.016	15.731	0.067		0.706	0.383	1.289	NS
Infection, yes		1.079	0.337	4.114	NS		NA	NA	NA	NA
Transfusion > 2 units of PRBCs		NA	NA	NA	NA		1.201	0.658	2.196	NS

## Discussion

Blood transfusion has always been a concern. It is true, blood transfusion does save many lives, especially in cardiac surgery where blood loss is often seen; however, blood is a scarce resource, and its complications need to be weighed against the benefits. Hence, there is a need for transfusion protocols. In critically ill patients, blood transfusion can greatly affect mortality [12]. Blood transfusion has also been seen to shift the outcome and prognosis in anemic patients [12,13]. In addition to these findings, there are many studies calculating the risk of morbidity and mortality in cardiac surgery patients receiving blood transfusion [8,14]. According to recent findings, centers are now taking up more conservative blood transfusion protocols [15].

A controversial risk associated with blood transfusion is post-operative infection. Although some papers have shown an association between blood transfusion and postoperative infection [8,16], our study showed no association. Our results, however, are not uncommon. Many other studies have found no relation between perioperative blood transfusion and postoperative infection in cardiac surgery patients [17]. In a prospective study by Ali et al., 232 cardiac surgery patients evenly divided into transfused and non-transfused groups showed no association between blood transfusion and postoperative infection. They have challenged the “opinion of withholding blood transfusion in patients solely because of concerns of predisposition to infection” [17]. In addition to blood transfusion, there are many independent factors that have been associated with an increase in post-operative infection [18]. Depending on the prevalence of those factors in a certain population and on the influence of those factors on postoperative infection, the results could vary greatly. Heart failure has been associated with an increased risk of post-operative infection, which is similar to the findings in our study [8].

The institution is a tertiary center with excellent blood storing and handling protocols. However, information such as time and reason for transfusion were not recorded. We practice a liberal blood transfusion protocol. Most blood is transfused intraoperatively and that is partly due to the drop in Hb that is seen during hemodilution when patients are placed on cardiopulmonary bypass. Our non-transfusion group was too small, which did not allow us to properly compare the two groups. Nevertheless, the association between blood transfusion and post-operative infection was clearly insignificant.

## Conclusions

After a thorough analysis of 197 patients undergoing a variety of cardiac surgical operations and with a multivariate analysis accounting for pre-operative factors, there was no association between the incidence of infection and blood transfusion. While there are other reasons for withholding blood, it would not be recommended to do so based on the concern of infection.

For future studies, we recommend a larger population and a prospective study design. We would also turn our attention to HbA1c and antibiotic prophylaxis and see how tight control on those factors would influence the incidence of postoperative infection in cardiac surgery patients.
